# Eosinophil-Derived Neurotoxin, Tumor Necrosis Factor Alpha, and Calprotectin as Non-Invasive Biomarkers of Food Protein-Induced Allergic Proctocolitis in Infants

**DOI:** 10.3390/jcm9103147

**Published:** 2020-09-29

**Authors:** Artur Rycyk, Beata Cudowska, Dariusz M. Lebensztejn

**Affiliations:** Department of Pediatrics, Gastroenterology, Hepatology, Nutrition and Allergology, Medical University of Bialystok, 15-089 Bialystok, Poland; beata.cudowska@umb.edu.pl (B.C.); dariusz.lebensztejn@umb.edu.pl (D.M.L.)

**Keywords:** infant, food allergy, fecal biomarkers, tumor necrosis factor α, eosinophil derived neurotoxin, calprotectin

## Abstract

Diagnosis of non-IgE mediated food allergy presents a special challenge due to lack of a single, non-invasive diagnostic method. We selected three fecal biomarkers of allergic inflammation of gastrointestinal origin in order to improve the diagnostic process. Twenty-seven infants with symptoms of hematochezia were prospectively enrolled into this study. All patients underwent a complete differential diagnosis of rectal bleeding. Non-IgE mediated food allergy was confirmed by an open, oral food challenge. The control group included twenty-five infants with functional gastrointestinal disorders. Eosinophil-derived neurotoxin (EDN), tumor necrosis factor alpha (TNFα), and calprotectin concentration were measured in stools of all children by enzyme-linked immunosorbent assays (ELISA) using commercial kits. Median eosinophil-derived neurotoxin and calprotectin fecal levels were significantly higher in the study group than in the control group (*p* < 0.05). The difference of fecal tumor necrosis factor alpha concentration between both groups was not statistically significant (*p* > 0.05). The best diagnostic performance was reached in a combination of fecal calprotectin (fCal) and EDN i.e., 88.9% and 84%, respectively. Fecal EDN and fCAl are reliable tools in differentiating between food protein-induced allergic proctocolitis and gastrointestinal functional disorders in infants.

## 1. Introduction

Food allergy (FA) is defined as an occurrence of reproducible, clinical symptoms caused by an abnormal, immune response to food components [1]. Based on the pathophysiologic background, immune hypersensitivity reactions to food are categorized into two main groups: IgE-mediated and non-IgE-mediated, with both requiring a different diagnostic approach.

The diagnosis of an IgE-mediated FA begins with a history of clinical symptoms, based on which skin pricks tests (SPT) and serum specific-IgE (sIgE) with potential allergens are carried out. Both methods present good sensitivity, but poor specificity [2]. Similarly, the application of a highly sensitive component-resolved diagnosis (CRD) is limited to immediate reactions exclusively [2]. What is more, the panel of sIgE presents an additional value in predicting polysymptomatic allergy development [3].

Non-IgE-mediated allergic reactions account for approximately 40% of cow’s milk protein (CMP) allergy in infants and young children. Broad clinical manifestations include food protein-induced enterocolitis syndrome (FPIES), food protein-induced allergic proctocolitis (FPIAP), food protein-induced enteropathy (FPIE), Heiner’s syndrome (pulmonary hemosiderosis) and CMP-induced iron deficiency anemia [4]. FPIAP is the most common entity, with a relatively prominent symptomatology of intermittent blood-streaked normal to moderately loose stools, which accounts for 18% to 64% of the infants with rectal bleeding [5,6].

Diagnosis of non-IgE-mediated FA is more challenging, because of the delayed onset of symptoms and blurred symptomatology. Since available data from the application of atopy patch tests (APT) are limited and indicate their poor sensitivity, they are not being recommended in the routine management [2]. Due to the lack of a single, reliable, diagnostic method, oral food challenge (OFC) remains the gold standard in FA [7].

Although most reliable, OFC has considerable limitations: time and resource consumption, potential risk of anaphylaxis, involvement of qualified medical practitioners, and stress for the patient [8].

In order to improve the management of FA, non-IgE in particular, alternative diagnostic methods have been studied: affinity of IgE binding, cytokines profile, T-cell number and function, B-cell activity, DNA methylation signatures, and so called “omics” which have already been shown to be useful in other gastrointestinal inflammatory diseases [9,10]. In comparison, fecal biomarkers are especially promising due to ease of collection, good correlation with the severity of inflammation in the intestinal wall (their place of origin), and specificity for intestine inflammation. Fecal calprotectin (fCal) has already found a reliable place in gastroenterology to diagnose and monitor inflammatory diseases [11]. Studies on pathophysiology of an allergic reaction have indicated the potential role of eosinophil-derived neurotoxin (EDN), representing the activation and degranulation of eosinophils and tumor necrosis factor α (TNFα) involved in the process of intestinal epithelial cell damage, increased intestinal permeability, and mucosal infiltration by leukocytes [12,13,14,15]. The combination of all three parameters might be a promising method in FA diagnostics. To the authors’ best knowledge, there has only been one study which targeted a similar biomarker profile, by Kalach et al. [16].

The aim of this study was to assess the usefulness of the simultaneous measurement of three non-invasive fecal biomarkers: EDN, fCal, TNFα in the diagnosis of non-IgE-mediated food allergy in children.

## 2. Materials and Methods

### 2.1. Patients and Study Design

**Figure 1:**
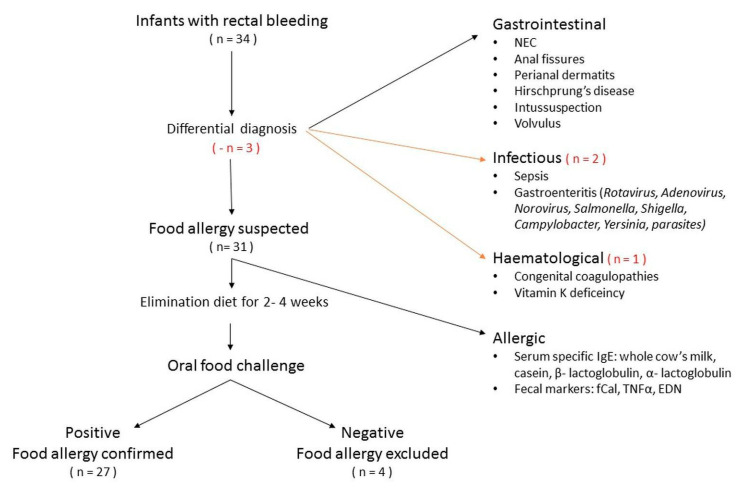
Study design. EDN, eosinophil derived neurotoxin; fCal, fecal calprotectin; NEC, necrotizing enterocolitis; TNFα, tumor necrosis factor α.

Thirty-one children aged between 1 and 12 months, admitted to the hospital with symptoms of hematochezia, were prospectively enrolled into the study. The presence of blood in the stool was verified by macroscopic evaluation or a positive occult blood test. All patients underwent the standard protocol for differential diagnosis of infancy rectal bleeding. Schematic diagnostic procedures are shown in Figure 1. Children were excluded if they were diagnosed with: perianal excoriations or anal fissures (physical examination), anatomical abnormalities of the gastrointestinal tract (abdominal sonography and/or radiography), history of abdominal surgery, coagulation disorders (prothrombin time [PT] ≥ 17 s, international normalized ratio [INR] ≥ 1.5, activated partial thromboplastin time [APTT] ≥ 60 s, platelet count < 100,000 × 10^9^/L), vitamin K deficiency (INR ≥ 1.4), parasitic or infectious gastroenteritis (positive stool analysis or culture), necrotizing enterocolitis (NEC) (combination of physical examination, laboratory tests, and imaging characteristics with Bell staging), or sepsis (procalcitonin [PCT] ≥ 2.0 ng/mL). None of the selected patients required endoscopy in order to exclude inflammatory bowel disease.

Further steps the in allergic work-up followed the current Guideline of The European Society for Paediatric Gastroenterology Hepatology and Nutrition (ESPGHAN) [7]. In order to exclude IgE-mediated mechanisms, the serum-specific IgE panel with food allergens was carried out, followed by fecal marker measurement i.e., calprotectin, EDN and TNFα. Afterwards, all children in the study group were introduced to a diagnostic elimination diet. Depending on the clinical manifestation, patients were given an elimination diet i.e., either extensively hydrolyzed milk formula (eHF), amino acid-based formula (AAF), or egg-free/cow’s milk protein free dietary restrictions for children and their breastfeeding mothers. After a hematochezia resolution, followed by an elimination period of 2–4 weeks, all patients were subjected to an open, OFC according to ESPGHAN protocol.

Simultaneously with the study cohort, twenty-five children matched by gender and age, diagnosed at the same hospital with functional gastrointestinal disorders (according to revised IV Rome criteria) were selected as the control group. Patients with functional gastrointestinal disorders were included, due to the fact that, such diagnosis requires exclusion of an organic background.

A written informed consent was obtained from the parents of all patients. The study protocol was approved by the Local Ethics Committee under the registration number R-I-002/106/2017.

### 2.2. Serum-Specific IgE Measurement

Antigen-specific IgE were measured using PolyCheck^®^ RAST tests (Biocheck GmbH, Münster, Germany) calibrated according to the manufacturer’s titers. The IgE cut-off value was accessed upon 0.15 kU/L. The main food allergens were tested: cow’s milk protein (f02), egg white and egg yolk (f01, f35), soybean (f14), rice (f09), peanut (f13), flour mix (fx10), hazelnut (f17), and codfish (f03).

### 2.3. Open Oral Food Challenge

The open milk OFC was conducted according to the ESPGHAN guidelines during a one-day hospital stay [7]. The challenge used a standard cow’s milk based infant formula. Stepwise increasing doses (1, 3, 10, 30, 100 mL) were given in 30-min intervals and the tolerance was monitored by parental record of the child’s symptoms. The challenge was discontinued when adverse reactions were noted. Infants without symptoms continued to receive the formula at home with doses appropriate for age. To recognize the delayed onset of symptoms, parents were contacted on day 5, or earlier if requested. The test was considered positive and the challenge discontinued if any of the initial symptoms recurred (i.e., bloody or/and mucousy stools). Infants with clinical manifestation continued on a CMP-free diet. Finally, CMP allergy was diagnosed as the resolution of symptoms on the elimination diet and their relapse during the challenge. In all cases, in which the challenge proved positive and mothers wished to continue breastfeeding, calcium supplements were prescribed (i.e., 1000 mg/day) with subsequent dietetic counseling.

The egg OFC was introduced to the patients with positive clinical history and no improvement after initial cow’s milk protein elimination diet. The egg OFC was conducted according to a three-level stepwise manner [17]. In STEP 0, patients were given approximately one quarter of a boiled egg yolk (i.e., 3.5 g), which contains about 1.8 mg of egg protein. The tolerance was monitored by parental record of the child’s symptoms, similar to the one described above. Patients who tolerated STEP 0 were subjected to low-dose OFC (STEP 1—Pumpkin cake containing one heated egg yolk i.e., 213 mg of egg protein), medium-dose OFC (STEP 2—Pumpkin cake containing 1/4 heated whole egg i.e., 1550 mg), and finally, high-dose (STEP 3—One scrambled egg i.e., 6200 mg).

### 2.4. Fecal Markers Measurement

Fecal samples were collected from the diaper immediately after defecation, as a part of allergic work-up, before introducing an elimination diet and OFC. fCal was tested on the same day. The remaining stool sample was frozen and stored at −20 °C until assayed. Frozen stool samples were thawed before analysis. Feces samples were weighed (15 mg) on an assay balance. Afterwards, a buffer solution (0.75 mL of 1:10 diluted WASHBUF (wash buffer concentrate) for TNFα measurement; or 1.5 mL of 1:2.5 diluted IDK Extract^®^) was added, and the sample was vortex-mixed for 10 min. The samples were centrifuged (1000 rpm, 5 min) and subsequently allowed to stand for approximately 10 min for sediment to settle. For analysis the amount of 0.1 mL per well was used. The feces samples were tested using an IDK^®^ Immunodiagnostic AG Bensheim Germany ELISA kit for EDN and TNFα. EUROIMMUN Medizinische Labordiagnostica AG Lübeck Germany ELISA kit was used for fCal detection. The detection limits for each parameter were the following: TNFα = 10 pg/mL, EDN = 0.164 ng/mL, fCal = 6.5 µg/g.

### 2.5. Statistical Analysis

Statistical tests, computing and graphics were performed using the STATISTICA 13 software (TIBCO Software Inc., Palo Alto, CA, USA). Variables with a normal distribution are expressed as means ± standard deviations (SD), whereas variables with a non-normal distribution are expressed as medians and ranges. In order to investigate whether the biomarker’s distribution is similar to the normal distribution, the Shapiro–Wilk test was performed. Differences between quantitative parameters were analyzed using the non-parametric Mann–Whitney U-test. Differences between qualitative parameters were calculated by the Χ^2^-test. The non-parametric Spearman’s test was employed for determining the correlations. Cut-off levels, specificity and sensitivity were calculated using the receiver operating characteristic (ROC) analysis. To determine the diagnostic usefulness of combined markers, synthetic indicators were developed. These indicators are linear combinations of selected variables. These synthetic indicators were used to construct ROC curves and calculate the area under the curves (AUCs). To calculate the sensitivity, specificity, positive predictive value, negative predictive value, and accuracy of synthetic indicators, cut-off values were selected, based on the criterion of maximization of Youden’s J statistic and the criterion of minimization of Euclidean distance to perfect classifier. *p*-value of <0.05 was considered statistically significant.

## 3. Results

Between December 2017 and March 2019, a total number of 59 children were enrolled into the study i.e., 34 infants with gastrointestinal bleeding and 25 patients with functional disorders as a control group. Standard diagnostic work-ups identified 2 children with acute gastroenteritis and one with Wiskott–Aldrich syndrome. OFC was negative in 4 patients (Figure 1). Detailed patients’ characteristics are provided in Table 1.

Patients diagnosed with FA encompassed 73.5% of all children with hematochezia referred to the hospital. The offending food was identified as CMP in 85% (23/27) of patients and hen’s egg in 15% (4/27) of cases. On follow-up, at approximately 12–16 months of age, 85% (23/27) of children gained tolerance to the allergen.

**Figure 2:**
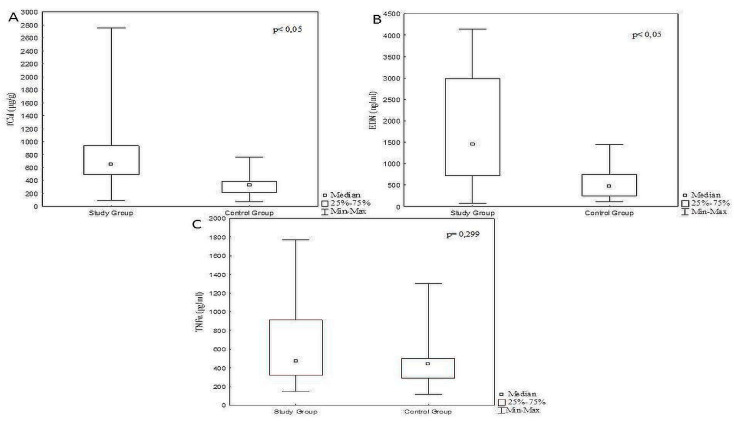
Differences in the concentration of fecal calprotectin (**A**), eosinophil derived neurotoxin (**B**) and tumor necrosis factor α (**C**) in infants with food protein induced allergic proctocolitis (study) and control groups.

Children with FA demonstrated significantly higher levels of fCal and EDN, compared with the controls (*p* < 0.05) (Table 1, Figure 2).

**Figure 3:**
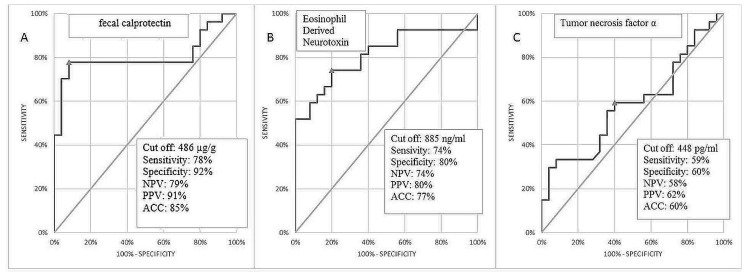
Receiver operating characteristics (ROC) curve of fecal calprotectin (**A**), eosinophil derived neurotoxin (**B**) and TNFα (**C**) for prediction of FPIAP. Abbreviations: NPV, negative predictive values; PPV, positive predictive values; ACC, accuracy.

In order to select the best marker’s combination for the discrimination of children with FA from control subjects, an ROC curve analysis was performed. Although the AUC values for fCal and EDN were above 0.8 (0.803 and 0.8119 respectively), the specificity of the test was the highest for fCal solely i.e., 92% for cut-off 486 ug/g (Figure 3).

The best diagnostic performance, regarded as significant increase in AUC, sensitivity, and specificity was reached in a combination of fCal and EDN (Table 2). Adding TNFα did not improve the diagnostic usefulness. Detailed statistical analyses of markers concentration in both groups are presented in Table 2 and Figure 3.

## 4. Discussion

This prospective study presents a potential role of three selected non- invasive, fecal biomarkers measurement in improving the diagnosis of FPIAP in children. The study provides the following new information: fCal and fecal EDN concentration proved to be significantly higher in children with FPIAP than with the gastrointestinal functional disorders and thus potentially useful in differentiating between two clinical entities; both biomarkers presented mutual correlation indicating simultaneous involvement of neutrophils and eosinophils in pathophysiology of FPIAP; a combined measurement of fCal and EDN presents better diagnostic performance than testing each biomarker solely; sensitivity and specificity of combined fCal and EDN testing reached 88.9% and 84%, respectively.

In relation to the current state of knowledge on FPIAP, the study cohort might seem unusual with relatively low breastfeeding percentage (37%), which is more typical for FPIES. Indeed FPIES, especially chronic type, might present only with a single reaction accompanied by bloody and/or mucous stools [18]. We retrospectively reanalyzed the study cohort and found no FPIES cases fulfilling the diagnostic criteria by Nowak-Wegrzyn et al. [19]. It can be speculated that the low number of breastfed children in our study might be a result of parental distress caused by lack of immediate improvement after initial, maternal, dietary restrictions, leading to introducing hydrolyzed milk formula.

A number of studies have shown that fCal is a sensitive marker for inflammation within gastrointestinal tract [20]. What is more, it has shown a good correlation with other inflammatory markers, such as plasma C-reactive protein (CRP) and erythrocyte sedimentation rate (ESR), providing additional value in diagnosis and management of patients with IBD 10. Ezri et al. indicated that fCal values are age-dependent and estimated cut-off levels upon <350 µg/g in the first year of life [21]. A recent study has presented the normogram for fCal concentration, in which the 95th percentile being 910.3 mg/kg for 0–12 months of age [22]. In our study, the median fCal value was 331.7 µg/g, 74–751 µg/g in 25 controls below 1 year of age, similar to Ezir et al. results and significantly lower than the Roca et al. study. Discrepancies might be a result of including neonates into the study group, in which fCal levels tend to be higher due to a more permeable small bowel [23]. The study by Oord and Hornung presented that the 97th percentile for fCal was 538 mg/kg in the group 1 to 6 months and 162 mg/kg in the group 6 to 12 months [24]. On the contrary, a recent study conducted in healthy infants aged 0–12 months revealed a median fCal concentration of 313 μg/g [25]. In our opinion, every arbitrary approach to age group will involve the risk of bias. What is more, intestinal epithelial homeostasis in children is particularly susceptible to the effect of intestinal microbiome, which may result in great variety of fCal concentrations [26]. Finally, different biomarkers’ concentration in studies might result from preanalytical errors i.e., various time of sample collection. Olafsdottir et al. reported that stool collected from the diaper had up to 30% higher calprotectin concentration due to water absorption [27]. In relation to allergy, Beser et al. revealed that in non-IgE mediated type, fCal tends to present significantly higher levels than in IgE-mediated ones (886 ± 278 μg/g vs. 392 ± 209 μg/g, respectively) [28]. In the presented study, patients with FPIAP demonstrated similar results of fCal concentration: median value 651.1 µg/g, 88.2–2755.4 µg/g. In a similar study group, infants with hematochezia and presumptive allergic colitis, Baldasare et al. described lower mean values of fCal (325.89 ± 152.31 µg/g) than in our study [29]. In a recent study, the mean fCal level in a group of 40 infants with cow’s milk protein allergy (CMPA) was 442 μg/g, whereas in the control group—100 μg/g, leading to the conclusion that optimal cut-off point for fCal should be 138 µg/g [30]. However, we suggest a cut-off point for fCal upon 485.65 µg/g, with the sensitivity of 77.8% and specificity of 92%.

Studies suggest the usefulness of fCal measurement in follow up in children with diagnosed CMPA [28,29]. However contrary evidence also exists [31]. An increased level of fCal has also been described in gastrointestinal malignancies, infections, polyps, and in nonsteroidal anti-inflammatory drug-therapies and therefore is not specific to allergic reaction [32].

During an effector phase of an allergic reaction, recruitment of mononuclear inflammatory cells leads to the release of a number of proinflammatory cytokines-including EDN [33]. Previous studies concerning children with atopic dermatitis and suspected food allergy revealed the usefulness of fecal eosinophil-derived proteins (eosinophil cationic protein [ECP], eosinophil protein-X [EPX]) in diagnostic workup [34]. Further studies by Wada et al. revealed fecal EDN concentration changes in response to control allergen stimuli in 8 patients with non-IgE-mediated food allergy [12]. Baseline levels of biomarker were variable, however after OFC, a significant increase was noted in all patients and a maximum concentration after 24 h (mean 33.244 ng/mL) of exposure. Among children with gastrointestinal allergy, those with hematochezia exhibited higher values of fecal EDN [13]. Kalach et al. studied a group of children with CMPA, in which fecal EDN in a single spot sample was measured [16]. Although fecal EDN did not differ significantly between both groups (*p* = 0.06), it was positively correlated with an allergic condition, with cut-off value for CMPA of 2818 ng/g, sensitivity of 54.5% and specificity of 85.7%. In our study, the mean EDN concentration in stool was significantly higher in the study group than in controls (*p* <0.05), sensitivity reached 74.1%, specificity 80% with cut off value 884.45 ng/mL. The before mentioned study, by Roca et al., estimated the 95th percentile for EDN upon 7.4 mg/kg in infants, which remains in the agreement with our results [22].

TNF-α is involved in pathophysiology of gastrointestinal allergy by initiating the process of increased intestinal permeability [35]. Although Wada et al. reported that levels of fecal TNF-α were not significantly elevated between patients positive to OFC and control group, they remained increased for one month’s time after the oral challenges [14]. In the presented study, a comparison of TNFα levels in both groups revealed no significant differences (*p* = 0.299). In the cohort of healthy children, TNFα levels below 90 pg/g were considered normal [15]. In our study, the median biomarker value in the control group was higher, reaching 443.6 pg/g, 120.5–1302.7 pg/g. In the case of allergic disorders, Majamaa et al. indicated that a particularly high concentrations of TNFα were found in patients manifesting delayed-onset allergy reactions [36]. However, Kalach et al. found fecal TNFα levels below detection range of 30 pg/g in all patients with CMPA [16]. The wide range of results might arise from the fact that TNFα is closely correlated with the inflammatory activity within intestine mucosa and susceptible to degranulation, which makes the timing of measurement critical [37,38]. It might be speculated that fecal TNFα measurement might be useful in differentiation between FA and inflammatory bowel disease in children.

Several limitations of the study require consideration. Firstly, only children below 12 months of age, with particular symptoms and diagnosed in hospital exclusively, were enrolled, which makes the study susceptible to selection biases, and thus not representative of the general population of patients with FA. Secondly, OFC was performed only in the study group, leaving potentially, subclinical cases of FA undetected and thus potentially interfering with the results. Thirdly, as blood carries neutrophils, bloody stools might overestimate the true value of fecal calprotectin resulting from allergic, gut inflammation. In addition, the cohort of patients might be considered as modest, however it is comparable to other studies of similar interest in the pediatric population. Moreover, a single spot biomarkers’ measurement allows only for the assessment of diagnostic usefulness, but not for the follow up process. Further studies in larger cohorts of patients are required in this field.

From a practical point of view, a fecal biomarkers measurement is cheaper, faster, and more patient-friendly than the standard diagnostic work-up. Simultaneous testing for fCal and EDN might differentiate patients with FPIAP from infants with, common in this age, gastrointestinal, functional disorders. Furthermore, it can possibly shorten diagnosis, as their laboratory measurement is faster than typical time of 72–96 h for resolution of symptoms. Moreover, due to their relatively simplicity in laboratory analysis, their testing might enhance differential diagnosis on outpatient’s basis, limiting unnecessary hospital admissions and lowering financial burden on health insurance systems. In doubtful cases (i.e., lack of improvement after initial treatment, polyvalent allergy suspected), fCal and EDN might support diagnostic decisions by avoidance furthers steps in differential diagnostics. In terms of science, fCal and EDN might be useful in research on pathophysiology of controversial conditions, like food protein induced constipation.

## Figures and Tables

**Table 1:** Background data, median (min-max; first, third quartile) values of fecal biomarkers among study and control groups. Significance is accepted at *p* < 0.05.

	Group		*p*-Value
	Study	Control	
Total number	27	25	NS
Age (median, months)	4	5	
(range; first, third quartile)	(1–11; 2, 6)	(2–10; 3, 6)	
Sex			<0.05
Girls	9	11	
Boys	18	14	
Primary diet			NS
Breast- fed	10	10	
Milk formula	17	15	
Biomarker			
fCal (µg/g)	651.1 (88–2755; 491, 934)	332 (74–759; 218, 384)	<0.05
EDN (ng/mL)	1450.8 (75.6–4146; 725, 2985)	471 (109–1446; 251, 749)	<0.05
TNFα (pg/mL)	472 (148–1772; 320, 913)	444 (121–1303; 288, 503)	NS
Treatment diet		N/A	
Maternal diet	7	–	
Casein eHF	10	–	
Whey eHF	1	–	
AAF	5	–	
Egg free diet	4	–	
Gained tolerance		N/A	
Yes	23	–	
No	4	–	

Abbreviations: AAF, amino acid formula; EDN, eosinophil derived neurotoxin; eHF, extensively hydrolyzed protein formula; fCal, fecal calprotectin; N/A, not applicable; NS, not significant; TNFα, tumor necrosis factor α.

**Table 2:** Area under the curve (AUC), standard error (SE), confidence interval, sensitivity (sen), specificity (spec) of eosinophil derived neurotoxin (EDN), tumor necrosis factor α (TNFα), and fecal calprotectin (fCal) among study group.

Variable	AUC	SE	95% C.I. (AUC)	*p*-Value (AUC = 0.5)	Minimum Euclidean Distance Classifier			Youden’s Index		
					Cut-off	Sen	Spec	Cut-off	Sen	Spec
EDN (ng/mL)	0.8119	0.0625	(0.689–0.934)	0.0000	>884	74%	80%	>884	74%	80%
TNFα (pg/mL)	0.5844	0.0809	(0.426–0.743)	0.2966	>448	59%	60%	>733	30%	96%
fCal (µg/g)	0.803	0.0687	(0.668–938)	0.0000	>486	78%	92%	>486	78%	92%
EDN/TNFα	0.8141	0.062	(0.693–0.936)	0.0000		74%	84%		67%	92%
EDN/fCal	0.8778	0.0524	(0.775–0.98)	0.0000		89%	84%		89%	84%
TNFα/fCal	0.8044	0.0693	(0.669–0.94)	0.0000		78%	96%		78%	96%
EDN/TNFα/fCal	0.8756	0.0539	(0.77–0.981)	0.0000		89%	84%		89%	84%

Abbreviations: AUC, area under the curve; C.I, confidence interval; EDN, eosinophil derived neurotoxin; fCal, fecal calprotectin; SE, sensitivity; Sen, sensitivity; Spec, specificity; TNFα, tumor necrosis factor α;.
